# Rapid prediction of single green coffee bean moisture and lipid content by hyperspectral imaging

**DOI:** 10.1016/j.jfoodeng.2018.01.009

**Published:** 2018-06

**Authors:** Nicola Caporaso, Martin B. Whitworth, Stephen Grebby, Ian D. Fisk

**Affiliations:** a Campden BRI, Chipping Campden, Gloucestershire, GL55 6LD, UK; b Division of Food Sciences, University of Nottingham, Sutton Bonington Campus, LE12 5RD, UK; c Nottingham Geospatial Institute, Faculty of Engineering, University of Nottingham, Innovation Park, NG7 2TU, UK

**Keywords:** Machine vision technology, Coffee quality, Chemical imaging, Coffee fat, Near-infrared spectroscopy, Individual bean analysis

## Abstract

Hyperspectral imaging (1000–2500 nm) was used for rapid prediction of moisture and total lipid content in intact green coffee beans on a single bean basis. Arabica and Robusta samples from several growing locations were scanned using a “push-broom” system. Hypercubes were segmented to select single beans, and average spectra were measured for each bean. Partial Least Squares regression was used to build quantitative prediction models on single beans (n = 320–350). The models exhibited good performance and acceptable prediction errors of ∼0.28% for moisture and ∼0.89% for lipids.

This study represents the first time that HSI-based quantitative prediction models have been developed for coffee, and specifically green coffee beans. In addition, this is the first attempt to build such models using single intact coffee beans. The composition variability between beans was studied, and fat and moisture distribution were visualized within individual coffee beans. This rapid, non-destructive approach could have important applications for research laboratories, breeding programmes, and for rapid screening for industry.

## Highlights

• Intact single green coffee beans were analysed for their moisture and fat content by HSI.
• NIR Hyperspectral imaging was applied to develop PLS calibrations for these constituents.
• The PLSR performance showed a performance comparable with traditional NIR instrumentation.
• A classification model was successfully applied by PLS-DA to discriminate Arabica vs Robusta.

## Graphical abstract

**Figure:**
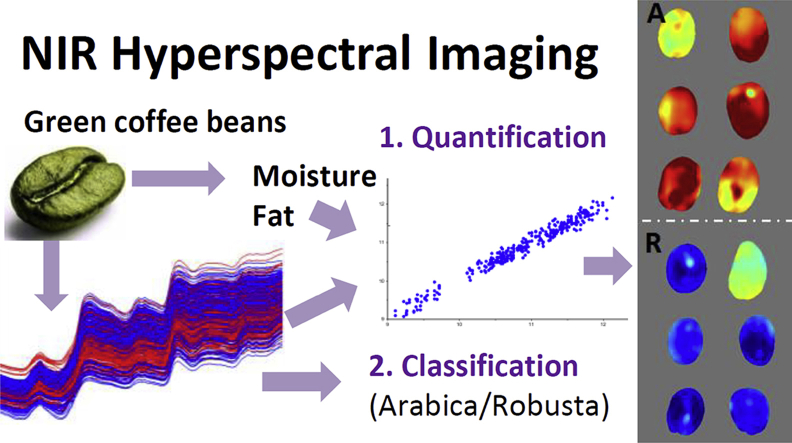

## Abbreviations

HSI: Hyperspectral Imaging
LDA: Linear Discriminant Analysis
LV: Latent Variable
MLR: Multiple Linear Regression
MSC: Multiple Scatter Correction
NIR: Near Infrared
NIRS: Near Infrared Spectroscopy
NMR: Nuclear Magnetic Resonance
PC: Principal Component
PLSR: Partial Least Squares Regression
RMSEC: Root-Mean Square Error of Calibration
RMSECV: Root-Mean Square Error of Cross Validation
RPD: Ratio of Performance Deviation
SNV: Standard Normal Variate
SVM: Support Vector Machine

## 1 Introduction

### 1.1 Coffee composition and quality

Coffee is one of the most popular beverages worldwide, and the quality of the final product is defined by several factors. Most of these strictly depend on the green bean composition (Illy and Viani, 2005), including moisture and fat, which are among the main constituents of green coffee beans. Moisture, in particular, is a critical quality parameter because it affects coffee bean shelf life. The determination of coffee bean moisture content is paramount to ensure safe transport and storage and to avoid the risk of mould development (fungal growth during storage), since an excessively dry or excessively wet green coffee bean will not maintain its quality (Wintgens, 2009). Lipids are another important component of green coffee beans, with fat content ranging from 7–10% in Robusta coffee and up to 15–17% in Arabica. The majority of coffee lipids are represented by triacylglycerols (75%), diterpene esters (up to 20%), sterols (2–3%), free fatty acids (1%) and tocopherols (0.05%) (Farah, 2012). Unsaturated fatty acids are the most abundant fatty acids in coffee beans, and they are relevant not only in terms of health effects and sensory impact of the final coffee brew, but also for the shelf life and storage of the raw material. Rancidity as a food defect comprises oxidative or hydrolytic rancidity, and is strongly influenced by the fatty acid composition and the total amount of fat present. Diterpenes, both free and esterified, form 0.2–1.2% of coffee constituents. Fat is primarily located in the endosperm of coffee seeds. There is also a layer of wax on their surface, accounting for 0.2–0.3% of the coffee weight (Farah, 2012).

Several methods are available for the measurement of moisture in coffee, including the oven drying method, Karl-Fischer titration, conductivity meters and water activity measurement (Reh et al., 2006). Conductivity meters are relatively rapid and easy to use but cannot measure a single coffee bean, and so alternative methods should be applied when focusing on individual coffee beans. Furthermore, the traditional solvent extraction techniques applied for fat analysis (e.g. Soxhlet method) are hazardous and time consuming and can take over 16h (Speer and Kölling-Speer, 2006). Rapid analytical techniques are therefore needed for the quantification of total lipids in foods.

The application of non-destructive techniques such as Near-Infrared Spectroscopy (NIRS) provides the means to analyse coffee beans rapidly without the need for organic solvents. In addition, contrary to other techniques, NIRS can be applied to solid samples without preparation, e.g. grinding, extraction or purification (Pizarro et al., 2004). Although NIRS is an indirect technique with high accuracy for moisture prediction in coffee beans, the need for good calibration has been highlighted (Reh et al., 2006). Despite being a powerful technique, NIRS does not readily permit detailed analysis in the spatial domain, which is key to understanding bean-to-bean variation and the spatial distribution of moisture and lipids across seeds. In common usage, NIRS is therefore limited to application to homogenous food samples.

### 1.2 Hyperspectral imaging for coffee analysis

Hyperspectral imaging (HSI) combines the non-destructive nature of NIR technology with image analysis, enabling the possibility of rapid analysis and screening of multiple grains at a time, and measurement of both physical features and chemical composition. In particular, “push-broom” sensors offer the potential to implement HSI both on a laboratory scale and for industrial application. By exploiting the spatial domain, the amount of information is dramatically increased with HSI, as each pixel of the image corresponds to a full spectrum in the NIR region (ElMasry et al., 2007). Consequently, HSI offers the advantage of visualizing the concentration of chemical components in whole intact food samples at the single pixel level and single object level (e.g. whole wheat kernels) once a proper calibration has been established. In this way, information on the distribution and variability within and between kernels is obtained (Caporaso et al., 2018a, Caporaso et al., 2017). However, the majority of applications of HSI in the food science sector concern classification methods rather than quantitative prediction models of composition. With respect to moisture and fat determination by HSI, very limited research has been published on grains and seeds, with the majority of studies reporting on other food products such as meat (Xiong et al., 2014). There is even less published research relating to HSI prediction of food constituents at single object level. Examples of HSI applied to foods for moisture prediction include the analysis of single strawberry fruits using the spectral range 400–1000 nm (ElMasry et al., 2007), and single peanuts using two spectral ranges (400–1000 and 1000–2500 nm) (Jin et al., 2015). Both peanut models return calibration and prediction coefficients of determination (R^2^) of about 0.9, with Root Mean Square Error of Calibration (RMSEC) and of cross validation (RMSECV) of 0.06%, for a moisture content ranging from 3.5 to 4.5%. A higher error was reported by Cogdill et al. (2004) for moisture prediction in maize seed kernels using HSI. Utilizing the 750–1100 nm spectral region, the reported calibration had R^2^ = 0.87 and prediction errors slightly above 1% for maize kernels with a moisture content between 9.7% and 30.5% and lipid content of 0.3–12.2% (“as is” basis).

Despite these few applications of HSI for quantitative predictions in granular food products, very little work has been published on coffee, and there is a complete absence of literature relating to single beans or green coffee bean quantitative models by HSI. Only a single study investigating moisture determination by HSI on instant granulated coffee has been published (Achata et al., 2015). The coffee moisture content was artificially changed at defined levels in the laboratory and scanned by HSI in the spectral region 880–1720 nm, showing excellent performance.

### 1.3 Hyperspectral imaging for classification purposes

Coffee species classification (i.e. Arabica and Robusta) is pertinent to the coffee industry because of their differing commercial price; Arabica is considered higher quality and more expensive than Robusta coffee (Rubayiza and Meurens, 2005). While coffee bean species identification is a relatively easy task for experts based on visual inspection of the green coffee, discrimination between Arabica and Robusta on roasted samples is a more challenging task (Keidel et al., 2010). Even for green coffee there is the need to assess individual beans within a whole batch, due to the variability expected within the population, caused by genetic, environmental and post-harvest factors. However, this is impractical to undertake through visual inspection and so rapid, objective methods are required.

Several analytical approaches have been explored to verify whether molecular markers exist to discriminate ground and roasted Arabica and Robusta coffees based on fat composition. For example, Romano et al. (2014) used fatty acid composition as a possible indicator to differentiate the two species, but the classification and quantification in blends is still a challenge as parameters such as the roasting degree strongly influence the lipid composition of the beans (Romano et al., 2014). However, these methods require grinding, extraction and time-consuming analytical techniques, e.g. gas-chromatography of volatile compounds, matrix-assisted laser desorption/ionization mass spectrometry or gas-chromatography-mass spectrometry (Myles et al., 2006). These methods are relatively complex, time-consuming, expensive, and are not applicable online (Buratti et al., 2015).

To address this, several authors have assessed the potential of non-destructive techniques for coffee bean discrimination, including NIRS (Downey et al., 1995, Myles et al., 2006). Good performance of NIRS for authenticity purposes of ground coffee has been demonstrated, e.g. for the quantification of other ground coffees in Kona coffee blends (Wang et al., 2009), the addition of barley in roasted coffee (Ebrahimi-Najafabadi et al., 2012), as well as extraneous materials intentionally added for fraudulent purposes (Barbin et al., 2014). Rubayiza and Meurens (2005) successfully classified coffee species using FT-Raman spectroscopy in the mid-infrared region based on green and roasted ground coffee beans. Reflectance NIRS in the region 1100–2500 nm was also successfully applied on ground and roasted coffees for the same purpose (Esteban-Diez et al., 2007). However, for unground coffee only a single example has been found in the literature, which used reflectance NIRS in the spectral region 1100–1800 nm in conjunction with Linear Discriminant Analysis (LDA), resulting in classification errors of 5% to 15% depending on the spectral pre-treatment applied (Myles et al., 2006). The only existing study to utilise HSI to discriminate Arabica and Robusta green coffee beans was recently published by Calvini et al. (2015). In this study, several classification methods were applied on the hypercube, obtaining an accuracy of 97%. The spectral region 955–1700 nm was utilised; the model was not built on a single coffee bean level.

From the above, it is clear that there has been very little attention on analysing coffee beans without prior grinding, and apparently no previous research dealing with quantitative prediction models of composition for green coffee based on HSI. In addition, calibrations for single coffee beans have not been previously reported for moisture and fat content, which can be of interest for breeders and the industry, to obtain information on the distribution of these constituents within the population via a non-destructive approach. Accordingly, the primary aim of the present study is to assess, for the first time, the application of HSI for total lipid content and moisture content prediction on green coffee beans on an individual seed basis. Moreover, a classification exercise to demonstrate a single-object approach for Arabica-Robusta species discrimination is also reported.

## 2 Materials and methods

### 2.1 Coffee samples

A total of 27 batches of green coffee beans were used for the experiment, and from each batch several coffee beans were randomly selected and individually analysed, so that the inter- and intra-batch variability was included. The samples were obtained from several producing countries, comprising Brazil, Colombia, Costa Rica, Ethiopia, India, Mexico, Honduras, Kenya, Nicaragua, Uganda, Rwanda and Vietnam. Both post-harvesting processing techniques were included, with approximately 60% wet processed and 40% dry processed. Twenty batches belonged to the Arabica species, while seven were Robusta. Approximately six beans were randomly selected from each batch for moisture and fat determination, while ten were selected for the species classification experiment. As the observed moisture range was relatively narrow, an extended moisture content range was also created artificially by treating two sub-batches of a Mexican Arabica coffee to obtain more dry and wet coffee beans. One sub-batch was placed in an oven at 50 °C, while a second one was placed in a chamber at saturated humidity, for 3 to 12 h.

To build Arabica-Robusta classification models, HSI was applied on both sides of beans both before and after roasting. The coffee beans were roasted using a Roastilino (Fracino, Birmingham, UK) roaster at 210 °C for 3 min, which resulted in a medium-high roasting degree.

### 2.2 Reference analyses for moisture and fat

Moisture content was analysed by oven drying using an adaption of the method ISO 11294:1994 (1994), which involved using a slightly lower temperature and longer time to avoid excessive sample degradation. This is because the analysis of moisture content through the official ISO method could result in a partial degradation of the product when analysing the coffee beans, which will influence the overall weight loss (Reh et al., 2006). The individual samples of green coffee beans were placed on an aluminium tray and dried in a Sanyo 112-F (San Diego, CA, USA) oven at 95 °C for about 24h. The water content was then expressed as percentage of coffee bean weight.

Nuclear Magnetic Resonance (NMR) was used for reference lipid analysis, due to its advantages in terms of rapidity and accuracy, and because the required sample size makes it feasible to analyse the single coffee beans. The single coffee beans were ground using a Retsch PM 200 Planetary Ball Mill grinder (Retsch, GmbH, Germany), by first cooling the sample using liquid nitrogen (cryomilling) and then grinding for 30 s at 25 min^−1^. The NMR-based fat analysis includes a complete drying of the sample ground material, followed by measurement of total lipid content using a CEM Smart Trac II Moisture and Fat analyzer (CEM Microwave Technology Ltd. Buckingham, UK). The instrument has a resolution of 0.01%, a fat range from 0.01% to 99.99% and a balance with 0.1 mg readability. The repeatability of the method was 0.24%.

### 2.3 Hyperspectral imaging and data treatment

Data was acquired using a laboratory-scale HSI system described by Caporaso et al. (2018a). The hyperspectral instrument was supplied by Gilden Photonics Ltd. (Glasgow, U.K.) and includes a SWIR spectral camera (Specim Ltd. Oulu, Finland) containing a cooled 14-bit 320 × 256 pixel HgCdTe detector and N25E spectrograph providing 256 spectral bands over a wavelength range of 980–2500 nm with a spectral resolution of ∼6 nm. Samples (intact beans) were presented on a movable sample stage illuminated using two 500 W incandescent lamps, and imaged using a push-broom approach. SpectralCube 3.0041 software (Specim) was used to control the camera and translation stage. A black reference measurement was obtained by recording approximately 100 frames after closing the camera shutter at the end of each scan, and a white reference was obtained using a white PTFE reference material (Caporaso et al., 2017, Millar et al., 2008). Hyperspectral images were acquired for the dorsal and ventral sides of the coffee beans, and were analysed using ENVI 5.2-IDL 8.4 (Harris Geospatial Solutions). Bad pixels and spikes in the images were first removed, and then object segmentation was carried out to select pixels corresponding to each of the coffee beans by thresholding the hypercube according to log_10_ (1/R_1186_) < 1, where R_1186_ is the reflectance at 1186 nm. The pixels belonging to each coffee bean were identified and an unweighted mean absorbance spectrum for each coffee bean was computed from the component pixel absorbance spectra and exported for subsequent statistical analysis.

### 2.4 Statistical analysis

Moisture and fat prediction in green coffee beans was carried out by Partial Least Squares regression (PLSR) analysis using The Unscrambler X 10.3 software (Camo, Norway). The log (1/R) spectra were processed to reduce scattering effects, using Standard Normal Variate (SNV), first or second derivatives using the Savitzky-Golay smoothing process, Multiplicative Scatter Correction (MSC), or de-trending and normalization (Rinnan et al., 2009).

Species classification models for green and roasted coffee beans were built using Linear Discriminant Analysis (LDA) and Support Vector Machine (SVM). The C-SVC SVM type was used for the classification, with several kernel types tested, including radial basis function and polynomial. To choose the best gamma and C values, a grid search was performed before calculating the SVM model, and a cross-validation was applied using 10 random segments.

LDA is a powerful tool that performs dimensionality reduction and automatic object classification. It is based on finding the optimal boundaries among classes, by maximising the between-class variance while minimizing the within-class variance. SVM is a non-linear modelling technique that finds the optimal hyperplane as a surface able to separate the largest fraction of datapoints, and it maximises the margins among classes. Detailed explanation on SVM classifiers, with examples applied to HSI, has been reported by Jiang et al. (2007). In the present case, LDA was performed using a linear method, assuming equal prior probability and applying a previous Principal Component Analysis (PCA). SVM classification was performed using a C-SVC type classifier with several kernel types tested, including radial basis function and polynomial.

The evaluation of PLS regression models for moisture and fat determination was performed by considering the coefficient of correlation (R^2^), and root mean square error for the calibration (RMSEC) and cross-validation (RMSECV) datasets. In addition, the Ratio of Performance to Deviation (RPD) was used to give an indication of the goodness of fit. This parameter is defined as the ratio between the standard deviation of the reference values and the RMSECV. Cross-validation was applied to evaluate the accuracy of the model, as RMSECV gives the uncertainty that can be expected for future prediction of unknown samples, based on the following equation:

$$RMSEC=\sqrt{\frac{\sum_{i=1}^{n_{p}}{\left({\hat{y}}_{i}-y_{i}\right)}^{2}}{n_{p}}}$$

ŷ_i_ being the predicted value for the sample i, y_i_ its measured value, and n_p_ the number of validated cases. The number of latent variables (LV) was chosen to minimize the RMSECV, by leaving the software to select the optimal number. The sample outliers were identified and removed according to the sample residuals and leverage, as well as on the Hotelling's T^2^ values (Fearn, 2002).

## 3 Results and discussion

### 3.1 Descriptive statistics for reference measurements

The average moisture content for green coffee beans at their natural moisture range was 10.8%, while the average total fat content was 14.7% and 16.4%, expressed on a “as is” basis and dry matter basis (dmb), respectively (Table 1). The values in Table 1a are derived from a single set of samples, whereas Table 1b reports the moisture content assessed by oven drying for samples in a “natural state” as well as for the batch of samples that was treated to consider a wider range of moisture content. As mentioned above, this was done to build a calibration with a moisture content extended beyond the narrow range of the samples received (SD = 0.8%). This is particularly important for considering cases where batches of coffee beans are stored in improper humidity conditions that can result in lower quality, and even be at risk from mould development. Generally a moisture content of between 8.0% and 12.5% is regarded as suitable for the storage of green coffee beans in order to avoid microbial growth and altered sensory quality.

**Table 1:** Descriptive statistics for reference measurements on green coffee beans for (**a**) fat prediction model and for (**b**) moisture models.

	Parameter	Mean	SD	Max	Min	Sample no.
a	Moisture by NMR (%)	10.28	1.33	17.23	7.35	352
	Fat (% "as is")	14.66	2.64	20.32	7.91	
	Fat (% dry matter basis)	16.41	3.10	22.74	8.13	
	Weight (mg)	119.00	37.10	252.40	32.60	
b	Moisture (oven drying) - natural	10.80	0.79	12.10	7.20	320
	Moisture (oven drying) - laboratory treated	14.86	10.77	52.10	4.50	480

The average total fat content in green coffee beans of Arabica and Robusta species was 17.51 ± 2.21% (average content dmb ± standard deviation) and 12.63 ± 1.85%, respectively; there was a statistically significant difference (p < 0.01, Student's t-test) between the two groups. Literature data for bulk measurements of green coffee report a range from 7% to 17% dmb, with strong differences depending on the species, i.e. 15% on average for Arabica and 10% for Robusta coffee (Speer and Kölling-Speer, 2006).

As expected, for the reference measurements no statistically significant difference was observed in moisture content of Arabica coffee batches compared to Robusta ones, despite obvious and statistically significant differences for total lipids, with Robusta having lower fat content than Arabica samples.

### 3.2 Moisture and total fat prediction models in single green coffee beans by HSI

**Fig. 1:**
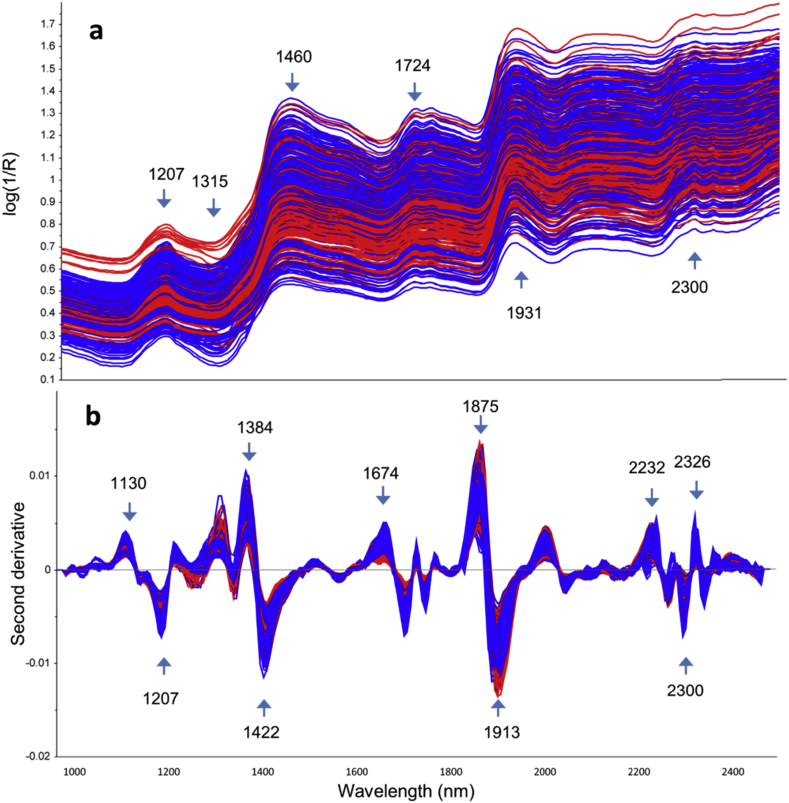
Mean reflectance spectra obtained from single green coffee beans: **a**) log (1/R); **b**) second derivative treatment. Red lines: Robusta; blue: Arabica. Numbers indicate the wavelength as nm.

The average reflectance spectra obtained for each coffee bean and the second derivative of these spectra are shown in Fig. 1, with the most characteristic spectral features indicated.

**Fig. 2:**
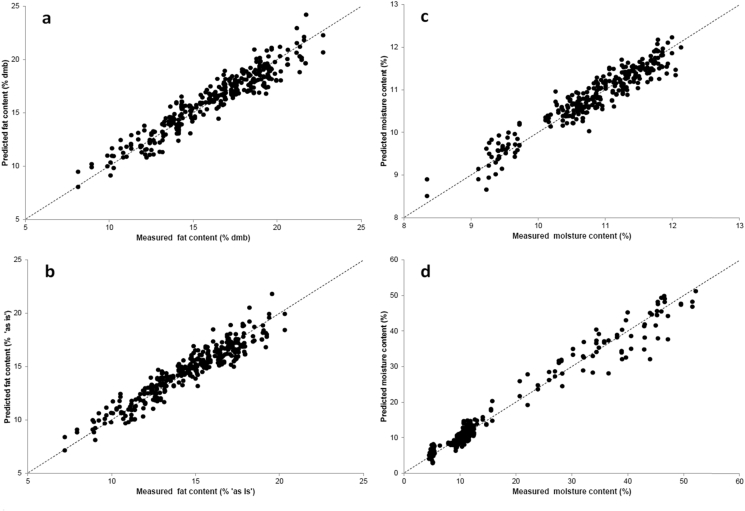
Prediction of coffee constituents in single green coffee beans using PLSR models based on HSI. **a**) Total fat content expressed as dry matter basis (dmb) (n = 345); **b**) Fat content expressed on “as is” basis (n = 345); **c**) Moisture content at the natural moisture range (n = 314); **d**) Moisture content on the extended moisture range (laboratory treated beans) (n = 463). The dotted line shows the ideal prediction.

The performance of the PLS regression models for moisture prediction by HSI built on individual green coffee beans was calculated using several spectral pre-treatment methods, which slightly influenced the final prediction error (Table 2). The first derivative treatment resulted in the best calibration performance for both sets of samples, with a calibration R^2^ (R_c_^2^) = 0.90 and cross-validation R^2^ (R_cv_^2^) = 0.86 in the case of natural moisture content, and R_c_^2^ = 0.97 and R_cv_^2^ = 0.96 for the laboratory treated beans. Considering the range of moisture content in both models, the obtained prediction errors are acceptable for quantification purposes, depending on the desired application: for natural moisture the prediction error was always much below 0.3% for a range of 4.2%, whereas for laboratory treated beans the cross-validation error was 2.0% for a moisture range of 47.6%. Plots of the four calibrations obtained are shown in Fig. 2.

**Table 2:** Performance of the PLS regression model for moisture content in single green coffee beans.

Parameter	Pre-processing	LV	R^2^ Cal	RMSEC	Slope	R^2^ Val	RMSECV	Slope	RPD
Natural moisture content	Log (1/R)	8	0.842	0.291	0.815	0.819	0.312	0.828	2.53
	Mean centered	8	0.844	0.289	0.844	0.825	0.307	0.834	2.57
	**First derivative**	**11**	**0.899**	**0.233**	**0.899**	**0.858**	**0.276**	**0.879**	**2.86**
	Second derivative	12	0.898	0.237	0.898	0.813	0.322	0.852	2.45
	SNV	12	0.897	0.235	0.897	0.850	0.285	0.872	2.77
Laboratory-treated beans	Log (1/R)	5	0.951	2.234	0.951	0.950	2.279	0.949	4.73
	Mean centered	5	0.947	2.314	0.947	0.947	2.372	0.946	4.54
	**First derivative**	**5**	**0.966**	**1.916**	**0.966**	**0.963**	**1.999**	**0.965**	**5.39**
	Second derivative	4	0.957	2.209	0.957	0.955	2.266	0.955	4.75
	SNV	3	0.962	2.041	0.963	0.912	2.074	0.962	5.19

Spectral range used: 980-2480 nm. LV: latent variable. SNV: standard normal variate spectral treatment. Error is indicated as %. RPD: ratio of performance deviation. Sample size (n): 320.

The capability of NIRS for moisture analysis in foodstuffs is widely known to be due to water molecules corresponding to strong absorbance at specific wavelengths in the near-infrared region. The application of NIRS has been reported by Osborne (1987) for moisture determination in flour, ground wheat and whole wheat, demonstrating a good performance of a multiple regression calibration built on the wavelengths of 1940 nm and 2310 nm. It was noted that whole kernels showed worse performance than ground material, with 0.29% prediction error in a moisture range 12.3–17.8%. The performance of our HSI moisture calibration is consistent with previous studies reporting HSI moisture calibration, when taking into consideration the prediction error and the range of reference moisture analysed (Cogdill et al., 2004, Jin et al., 2015). However, a direct comparison with these studies is not possible as they involve different granular foods and not coffee.

The results for total fat calibration in intact single green coffee beans are reported in Table 3. The second derivative pre-treatment gave the best model performance on an “as is” basis, obtaining a R^2^ value of 0.89 and 0.88 for the calibration and cross-validation datasets. When the lipid content was expressed on a dry matter basis, the R^2^ value was slightly better, being 0.90 and 0.89 for the calibration and validation datasets. The prediction error was approximately 1% in all cases, whether expressed on an “as is” or dry matter basis. In both cases, the cross validation error was below 1.0%, and thus perfectly suitable for quantitative purposes, considering the range of total fat (13.2% on “as is” basis). The second derivative treatment has also been successfully applied by other authors to analyse fat content and oxidation in food products through NIRS (Khodabux et al., 2007).

**Table 3:** Performance of the PLS regression model for total lipid content in green coffee beans expressed on an “as is” or dry matter basis.

	Pre-processing	LV	R^2^ Cal	RMSEC	Slope	R^2^ Val	RMSECV	Slope	RPD
As-is	Log (1/R)	6	0.850	1.019	0.850	0.837	1.062	0.843	2.49
	First derivative	4	0.866	0.961	0.866	0.860	0.985	0.857	2.68
	**Second derivative**	**6**	**0.893**	**0.861**	**0.893**	**0.883**	**0.904**	**0.885**	**2.92**
	SNV	5	0.859	0.990	0.859	0.849	1.028	0.857	2.57
	Baseline	6	0.852	1.010	0.852	0.841	1.050	0.847	2.51
Dry matter basis	Log (1/R)	6	0.864	1.087	0.864	0.855	1.125	0.862	2.76
	First derivative	4	0.871	1.062	0.871	0.866	1.091	0.864	2.84
	**Second derivative**	**6**	**0.900**	**0.935**	**0.900**	**0.890**	**0.985**	**0.890**	**3.15**
	SNV	5	0.864	1.081	0.864	0.855	1.117	0.860	2.78
	Baseline	6	0.857	1.116	0.857	0.845	1.169	0.853	2.65

Spectral range used: 980-2480 nm. LV: latent variable. SNV: standard normal variate spectral treatment. Error is indicated as %. Sample size (n): 350.

**Fig. 3:**
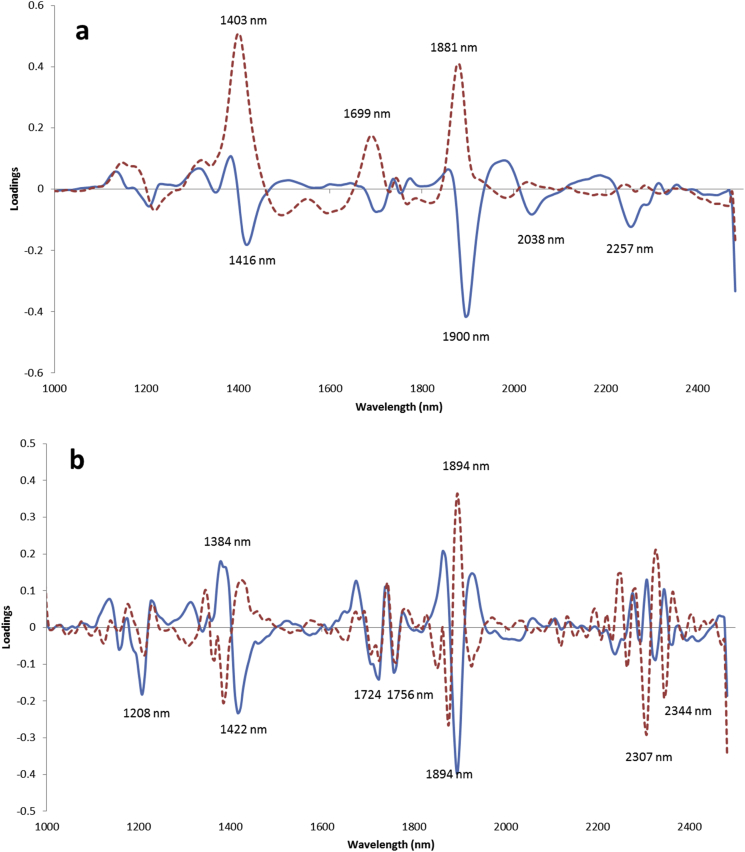
Loading of the first two PLS components for the (**a**) moisture (natural water content) and (**b**) fat (“as is” basis) PLS models showing the best performances for average spectra on a single green coffee bean basis obtained by HSI. The first (a) and second (b) derivatives were applied, respectively. Continuous blue line: PC1; dotted red line: PC2.

The loading plots for the best PLS regression models for moisture and total fat content are shown in Fig. 3. The strongest features observed for the moisture model are at 1416, 1900, 2038 and 2257 nm for PC1, and 1403, 1699 and 1881 nm for PC2 (Fig. 3a). For total fat, the highest loadings were for 1208, 1384, 1422, 1724, 1756, 1894, 2307 and 2344 nm (Fig. 3b). Previous research on water loss for green coffee under drying conditions described a major peak at 1940 nm (Reh et al., 2006), related to the 2nd overtone of O-H (Esteban-Diez et al., 2004). Morgano et al. (2008) reported that the most important wavelengths for NIR moisture prediction in green coffee were 1975, 1852, 2040 and 2150 nm, and their regression model allowed moisture quantification with a validation R^2^ of 0.818 and RMSECV 0.298%. Our results show comparable, but even better performance (R_cv_^2^ = 0.858 and RMSECV = 0.276%) despite employing a HSI approach on intact coffee beans, as opposed to using a NIR instrument on ground coffee material as in Morgano et al. (2008). In comparison, Cogdill et al. (2004) reported a poorer performance for moisture prediction model, with their best PLS regression model having R^2^ of 0.56 and a prediction error above 1%. The authors commented that this poor performance may be attributed to error both in the hyperspectral imager and the higher error obtained for the reference measurement, due to the fact that when single kernels/beans are analysed, they are destroyed with no possibility of averaging repeated measurements to decrease noise and to detect anomalies. They also analysed oil content but suggested using more accurate reference methods than traditional solvent extraction – specifically NMR – when analysing single seeds.

The peaks at 1210, 1360, 1700–1760 and 2275–2300 nm have been reported to arise from the first and second overtone of C-H, and stretching of the -CH2 groups. Absorption peaks around 1160 and 2130 nm were attributed to -HC=CH-, while the bands around 1200, 1400, 1750, 2310 and 2340 nm are usually associated to the C-H bond. The region between 2083 and 2222 nm is considered to be the combination of C-H stretching related to cis double bonds in the molecules, which exist due to the unsaturation of fatty acids (Khodabux et al., 2007).

The Ratio of Performance to Deviation (RPD) provides an indication of the quality of calibration equations for PLS regression models. Values above 2 are indicative of excellent models, whereas an RPD of between 1.4 and 2 is fair, and values below 1.4 indicative of non-reliable models (Ncama et al., 2017). In our case, the RPD was 2.9 for moisture with the natural content and 5.4 for the model with extended moisture range (see Table 2). This dramatic improvement for the laboratory treated beans was attributed to the very large range of moisture content, although the performance of the natural moisture content model suggests that it can be used for standardisation of the batch in order to detect single beans with excessive or very low moisture content.

The RPD value was 2.92 for lipids expressed on an “as is” basis, but the dry matter basis (dmb) model was more accurate with an RPD of 3.15. It is higher than the RPD values recently reported in our previous work for the prediction of sucrose, trigonelline and caffeine, where the latter compound showed RPD of 2.7 (Caporaso et al., 2018b). The performance of our model is comparable to other calibrations built using traditional NIR instruments, even for ground coffee beans. For instance, Pizarro et al. (2004) reported R^2^ calibration values of 0.763–0.987 for a PLS regression calibration for total fat in roasted and ground coffee using NIR data in the region 1100–2500 nm for a variety of spectral pre-treatments. However, it should be noted that roasted and green coffees have very different spectra, with also a dramatic change of the lipid content. The two species overlap in terms of fat content in the middle region of the distribution, for this reason a general model was proposed, which can be usefully applied to any coffee species. In addition, multivariate prediction models were separately built for the Arabica and the Robusta samples, obtaining a general improvement of the prediction error of approximately 0.15%. However, this slightly better prediction might not compensate for the disadvantage to have separate models, especially when blends are analysed.

### 3.3 Wavelength selection

For practical applications, data reduction strategies are of interest as they may reduce computer processing demands or enable cheaper multispectral sensors to be used to target fewer specific diagnostic wavelengths. To evaluate this, a wavelength reduction strategy was applied to the best models for moisture and fat prediction. The selection of the most important wavelengths was carried out based on thresholding the β-coefficients for these PLS regression prediction models. This approach assumes that only those wavelengths with large β-coefficients are useful for the prediction. Therefore, by setting a cut-off value, those bands with low influence on the full PLS regression model are removed and a new model is then built on a reduced number of spectral variables (Osborne, 1987). The threshold on the β-coefficients was varied to first determine the minimum number of wavebands that could be utilised without significant loss of prediction capability; resulting in selection of 42 bands for moisture and 22 for fat. Subsequently, the threshold was increased further in order to select only the six most important bands, to enable the prediction capability of a lower-cost multispectral sensor to be assessed.

Table 4 shows the performance of Multiple Linear Regression (MLR) models built using the selected wavelengths. The use of 42 bands led to a good performance compared to the PLS regression model reported in the previous section for moisture using the full spectra range. In fact, it had a RMSE of 0.242% and RMSECV of 0.286%, which is comparable to the PLS regression model. A further reduction of the number of selected wavelengths down to six still led to an R^2^ value of 0.615 and 0.596 for calibration and cross-validation, respectively, along with a marked increase in prediction error. It should be noted that this prediction is made on the reflectance data with no spectral pre-treatment, as derivatives cannot be applied in such circumstances, which brings a larger prediction error when using filter instruments with few selected wavelengths. Considering the fewer number of wavelengths utilised and the consequent advantages in terms of computational capacity required, the six band model could be still considered as acceptable for some practical applications for rapid screening of higher or lower moisture beans, and when lower cost sensors are needed.

**Table 4:** Multiple Linear Regression (MLR) models for moisture and fat content (% “as is” basis) in green coffee beans obtained by selecting the most important variables using log (1/R) values with no spectral pre-treatment.

Parameter	Pre-treatment	Spectral variables nr.	Calibration		Cross-validation		Validation Offset
			R^2^	RMSE	R^2^	RMSECV	
Moisture	log (1/R)	6^a^	0.615	0.459	0.596	0.471	0.61
		42	0.887	0.242	0.842	0.286	0.29
Fat		6^a^	0.693	1.464	0.676	1.503	4.57
		6^b^	0.841	1.053	0.834	1.078	2.36
		22	0.860	0.988	0.832	1.081	2.17

a Variable selection was performed based on the β-coefficients of the PLS model (filter method).

b Model built by PLS regression (LV = 6).

The fat prediction models were similarly affected by the number of variables selected, and the statistics applied. The use of 22 wavelength variables resulted in a performance almost identical to the PLSR model built using the full spectral range. This finding is in agreement with other studies (Xu et al., 2016), and suggests that most bands exhibit redundant information due to collinearity, and that waveband reduction techniques could be advantageous for the implementation of HSI technology for practical applications, e.g. screening of coffee bean fat content in the food industry. Reduced bands resulted in similar prediction error when using PLS regression, while the poorest overall performance was obtained using 6 wavelengths and MLR model. However, even in this case, the cross-validation error was 1.5%, which might be acceptable for some practical application and screening.

Despite water having characteristic absorption features in specific regions of the NIR spectrum (i.e. mostly where the O-H water bands absorb), the sole use of those wavelengths does not lead to good predictive models and so additional wavelengths are required. This is in agreement with the findings of Zhao et al. (2017), who recently applied HSI to predict fat and moisture content in ground beef using a limited number of wavelengths. They selected a higher number of variables for the moisture model, i.e. from 11 to 43, than the one to describe fat content (from 7 to 40), depending on the spectral pre-treatment applied.

In certain cases, wavelength reduction can even lead to higher prediction performance compared to the use of full spectra, probably because the uninformative bands are removed therefore reducing the noise in the prediction model. It should be also noted that using a PLS regression model with fewer bands can also allow more rapid prediction for the lower computation time required.

### 3.4 Application of the PLS calibrations and visualisation of compound distribution

**Fig. 4:**
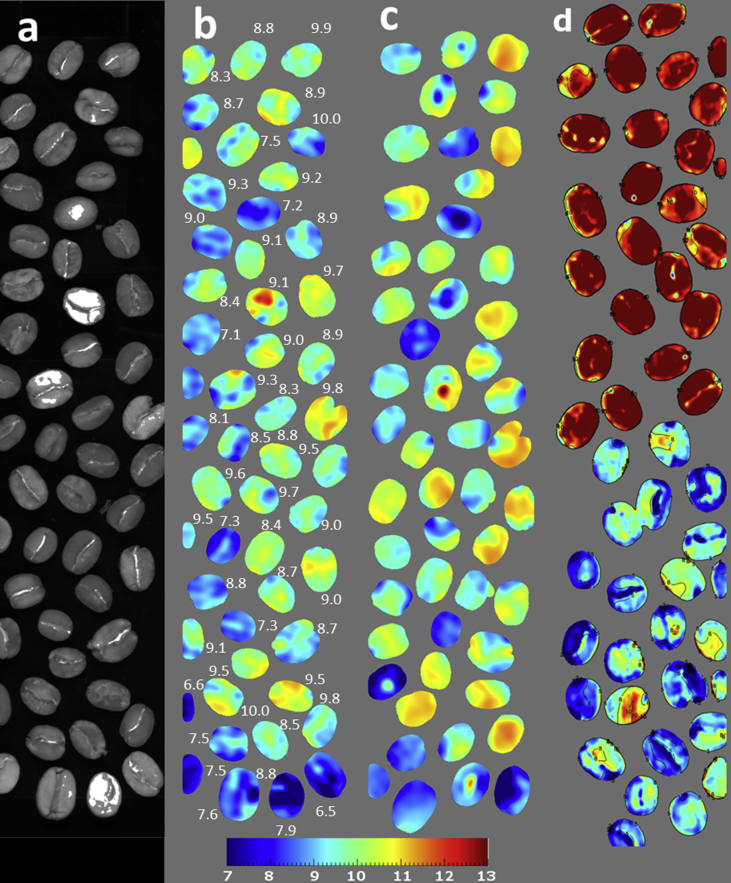
HSI calibration applied for moisture content in green coffee beans on a batch of Mexican Arabica**. a**) grayscale images showing the reflectance hypercube obtained from a continuous moving stage, at one spectral band; **b**) reconstructed image showing predicted moisture content visualized at single pixel level; **c**) predicted moisture obtained after the rotation of the coffee beans on the other face; **d**) batch subdivided into an aliquot placed under humid conditions (upper beans) or partially dried in an oven (lower). Numbers indicate the average predicted moisture expressed as %.

The devised PLS regression models for the prediction of moisture and total fat in green coffee beans were applied to HSI hypercubes to visualize the moisture and lipid distribution and content both between coffee beans and within individual beans. Fig. 4 shows the distribution and content of moisture in a batch of Mexican Arabica green coffee beans. By applying the calibration coefficients at a single pixel level, it was possible to both predict the average moisture content for a single coffee bean, and even visualize the water content within the beans. For some beans the distribution of moisture appears to vary somewhat throughout. However, there are practical difficulties in validating such differences at a single pixel level with the aid of analytical reference measurements.

**Fig. 5:**
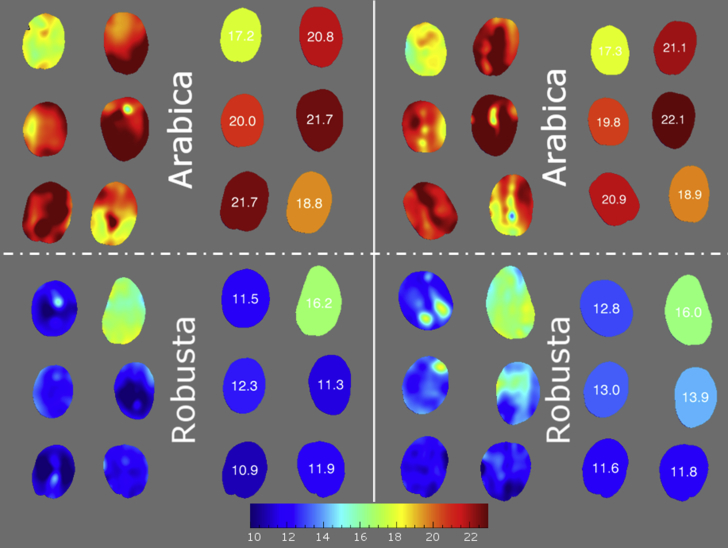
Application of HSI calibration (chemical imaging) for total lipid content visualisation in green coffee beans: example for Arabica (top) and Robusta (bottom) batches. The left and right halves of the figure show the same coffee beans placed on the opposite surface (inverted 180° about the y-axis). The numbers shown are the predicted average lipid content calculated from the pixel values for each bean, expressed as % (dmb).

Fig. 5 shows two batches of coffee beans, one Arabica and one Robusta, which clearly reveals the interspecies variation when visualizing the lipid distribution on a single pixel level. As expected, the Arabica and Robusta coffee batches had significant differences in their lipid content; the latter containing much lower fat. Minimal effects of coffee bean orientation were found during imaging. The six coffee beans belonging to the Arabica batch had lipid content ranging from approximately 17% to more than 22% (dmb). The Robusta batch ranged from approximately 11% to 16%. A calibration for fat such as this would be interesting not just for visualizing the distribution within individual coffee beans, but also for application to botanical and plant physiology studies related to lipid accumulation and especially the changes of lipid content at the outer layer in the post-harvest processing.

### 3.5 Coffee bean species classification

In addition to quantitative models, HSI is also applicable for rapid classification of intact granular food commodities, through the building of classification models. Both green and roasted beans (treated using the same time-temperature profile during roasting) were analysed individually. As reported in Table 5, for green coffee Linear Discriminant Analysis (LDA) produced better performances than Support Vector Machine (SVM), achieving up to 100% classification accuracy. The highest SVM classification accuracy for green coffee beans was just over 97%. For the roasted coffee beans, SVM achieved validation accuracies of 91.8 and 97.1% depending on the function applied, with SNV pre-treatment resulting in the best performance. For roasted coffee, LDA produced the best results with a linear function, achieving 100% correct classification accuracy for log (1/R), first and second derivative pre-treatment.

**Table 5:** Coffee bean classification models for species identification (Arabica-Robusta) based on Linear Discriminant Analysis (LDA) and Support Vector Machine (SVM) for green and roasted whole coffee beans. Sample size = 510 for green; 340 for roasted coffee.

Classification method	Function	Pre-treatment	Green		Roasted	
			Correct (%)	Incorrect (%)	Correct (%)	Incorrect (%)
LDA	Linear	Log (1/R)	98.39	1.61	98.53	1.47
		1st derivative	99.29	0.71	100.00	0.00
		SNV	98.57	1.43	98.53	1.47
		MSC	98.75	1.25	98.53	1.47
		2nd derivative	99.61	0.39	100.00	0.00
	Quadratic	Log (1/R)	100.00	0.00	100.00	0.00
		1st derivative	99.80	0.20	99.12	0.88
		SNV	99.80	0.20	99.71	0.29
		MSC	100.00	0.00	99.41	0.59
		2nd derivative	99.80	0.20	98.82	1.18
Classification method	Function	Pre-treatment	Training accuracy (%)	Validation accuracy (%)	Training accuracy (%)	Validation accuracy (%)
SVM	Polynomial	Log (1/R)	74.90	74.12	87.06	84.42
		1st derivative	72.55	72.55	64.71	64.71
		SNV	88.43	87.06	94.12	91.76
		MSC	82.75	82.16	95.00	88.24
		2nd derivative	72.55	72.55	64.71	64.71
	Radial basis function	Log (1/R)	84.90	83.92	90.29	88.24
		1st derivative	91.18	91.18	88.24	88.24
		SNV	97.45	97.25	98.23	97.06
		MSC	94.51	94.12	95.00	94.12
		2nd derivative	89.02	88.82	84.71	84.42

**Fig. 6:**
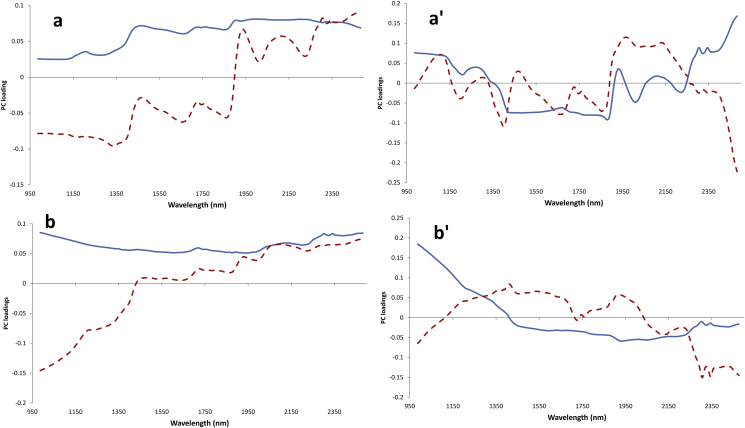
Loading plots from LDA for the Arabica-Robusta classification models, in single (left) green and (right) roasted coffee beans. **a**,**b**) log (1/R); **a'**,**b'**) MSC treated spectra. Continuous line: PC1; Dotted line: PC2.

The loadings for the LDA models for Arabica-Robusta discrimination on green and roasted coffee beans are shown in Fig. 6. The first PC for green coffee beans does not have particularly strong loading values at any specific wavelengths, whereas the plots for PC2 closely resemble the mean absorbance spectra (as seen in Fig. 1). For the roasted coffee beans, the same effect was observed for PC1, while PC2 had strong absorbance bands around 2000 nm, in addition to strong absorbance features around 1750 and 2350 nm where lipids mostly absorb.

This is the first study of Arabica-Robusta classification based on HSI using the full NIR spectral range. Previous publications reported on the possibility to group Arabica-Robusta beans by applying Principal Component Analysis (PCA) and to separate the two sets using the first two principal components (Esteban-Diez et al., 2007, Rubayiza and Meurens, 2005). The discrimination reported by Rubayiza and Meurens (2005) using FT-NIR was claimed to be mainly based on cafestol and kahweol, which the authors attributed to wavenumbers of 1567 and 1478 cm^−1^.

## 4 Conclusions

Hyperspectral imaging has been applied for the first time for the prediction of coffee constituents on a single coffee bean basis. The devised approach uses averaged spectral data from the hypercube to enable PLS calibrations to be built on a single object (bean) basis. We also demonstrated the wide distribution of lipid content within the same batch and between batches, and developed a moisture calibration that is capable of detecting problematic seeds within the population. Applying the calibration at a single pixel level provided the means to visualize compound distribution within individual coffee beans.

Our approach showed excellent prediction capabilities for both moisture and total fat content analysed through HSI, and this technique offers potential as a rapid and non-destructive method to obtain accurate indication of the coffee bean composition and uniformity in a whole bean dataset. Moreover, once calibrated, HSI can predict the moisture (and fat) content in a matter of seconds, compared to several minutes required for convention techniques such as a Rapid Moisture Analyzer. The potential of HSI for rapid, non-destructive screening of green coffee has great potential for research laboratories, plant physiologists and geneticists in plant breeding programmes, as well as for the food industry for quality control purposes.

## Declaration of conflict of interest

The authors declare no competing financial interest.
